# EMCN is associated with vascular-immune crosstalk and represents a potential biomarker in lung adenocarcinoma

**DOI:** 10.3389/fmolb.2026.1752442

**Published:** 2026-08-12

**Authors:** Yan Zhuang, Qingfeng Wang, Kaimin Wei, Fangjuan Wei, Xiaowei Yao, Yongliang Wang

**Affiliations:** 1 Department of Oncology Department, The People’s Hospital of Anyang, Anyang, China; 2 Department of Thoracic Surgery, The People’s Hospital of Anyang, Anyang, China

**Keywords:** biomarker, EMCN, immune infiltration, lung adenocarcinoma, machine learning, vascular-immune crosstalk

## Abstract

**Background:**

While MUC family genes have been established as prognostic biomarkers in gastric cancer, and GWAS studies link EMCN mutations to chemotherapy-induced myelosuppression in NSCLC, the systematic characterization of EMCN in lung adenocarcinoma (LUAD) remains elusive.

**Methods:**

This multi-omics strategy combining bulk and single-cell transcriptomics study integrated differential expression analysis, WGCNA, and machine learning algorithms (LASSO/SVM-RFE/Random Forest) to identify EMCN as a diagnostic hub gene, followed by experimental validation using immunohistochemistry Western blot and qRT-PCR.

**Results:**

EMCN (Endomucin) is a sialomucin-like glycoprotein predominantly expressed in vascular endothelial cells. Using bulk transcriptomic datasets and single-cell RNA-seq analysis, we found that EMCN expression was reduced in lung adenocarcinoma (LUAD) compared with non-tumor controls and was primarily localized to the endothelial compartment. Survival analysis using the median expression cutoff showed that high EMCN expression was associated with improved overall survival (Cox HR_high vs. low = 0.73, p = 0.04), indicating that low EMCN expression correlates with poorer prognosis. Machine learning-based feature selection (LASSO, Random Forest, and SVM) further prioritized EMCN among consensus candidate genes, supporting its potential relevance to the vascular-associated tumor microenvironment in LUAD. EMCN expression levels also showed a significant positive correlation with the degree of immune cell infiltration. Gene set enrichment analysis (GSEA) revealed that high EMCN expression in tumor tissues activates negative regulatory pathways associated with angiogenesis. Receiver operating characteristic (ROC) curve analysis highlights EMCN’s excellent diagnostic potential for LUAD, with an area under the curve (AUC) of 0.963. *In vitro* experiments confirm the downregulation of EMCN at both protein and mRNA levels, consistent with our bioinformatics predictions.

**Conclusion:**

This first comprehensive study establishes EMCN as a dual-functional regulator of vascular-immune crosstalk in LUAD, providing both a molecular diagnostic tool and therapeutic target for precision oncology.

## Introduction

Lung adenocarcinoma (LUAD) represents the most lethal thoracic malignancy, accounting for over 40% of global lung cancer deaths (Zha et al., 2024; Borg et al., 2024). While genomic profiling has identified driver mutations in EGFR/KRAS/ALK (Cai et al., 2023), the molecular heterogeneity underlying treatment resistance remains poorly characterized. This knowledge gap is particularly pressing given the limited 5-year survival rates (<20%) in advanced-stage patients receiving current targeted therapies (Tsuboi et al., 2023).

Emerging evidence implicates endothelial cell-derived factors in modulating tumor immune microenvironments (Guelfi et al., 2024). Notably, the endothelial mucin EMCN was recently associated with chemotherapy-induced myelosuppression in NSCLC (Huang et al., 2025), yet its mechanistic role in LUAD progression remains enigmatic. Specifically, three critical questions persist: (i) Is EMCN expression dysregulated in LUAD? (ii) Does it interact with immune infiltration patterns? (iii) Can it serve as a theranostic biomarker?

Previous attempts to identify LUAD biomarkers predominantly relied on single-omics approaches (Wei et al., 2024), failing to capture the complex crosstalk between tumor vasculature and immune evasion. Although GWAS studies linked EMCN variants to drug toxicity (Huang et al., 2025), these analyses lacked functional validation and spatial context. Moreover, conventional differential expression methods often overlook non-linear relationships detectable through advanced machine learning (Swanson et al., 2023).

Here, we integrated multi-omics bioinformatics combining bulk and single-cell transcriptomics to systematically decode EMCN’s clinical significance. Our workflow combines: (i) WGCNA for co-expression network construction (Langfelder and Horvath, 2008), (ii) ensemble machine learning (LASSO/SVM-RFE/Random Forest) for feature selection (Zhao et al., 2025; Li et al., 2024; Zheng et al., 2024), and (iii) CIBERSORT-based immune deconvolution (Chen et al., 2018; Guan et al., 2022). Crucially, we validate predictions through IHC and *in vitro* assays, establishing a translational pipeline from computational discovery to experimental verification.

In the present study, we discovered that endothelial cell adhesion molecule (EMCN) possesses dual characteristics of vascular and immune regulation. Additionally, EMCN exerts a negative regulatory effect on angiogenesis within tumors. We further validated the expression specificity of EMCN in patient tissues. Cox regression analysis revealed a significant correlation between EMCN expression and overall survival (OS) in patients with lung adenocarcinoma (LUAD). Therefore, we propose that EMCN can serve as a potential prognostic biomarker for LUAD.

## Materials and methods

### Datasets

Our study leverages the GSE31210 dataset from the Gene Expression Omnibus (GEO) database (http://www.ncbi.nlm.nih.gov/geo), which comprises gene expression profiles of 226 frozen lung adenocarcinoma (LUAD) tissues and 20 corresponding frozen normal lung tissues (Okayama et al., 2012). Additionally, data from The Cancer Genome Atlas (TCGA) and the Genotype-Tissue Expression (GTEx) projects were utilized for pan-cancer analysis. For comprehensive analysis of hub genes and their associations with clinical indicators, the Gene Expression Profiling Interactive Analysis 2 (GEPIA2, http://gepia2.cancer-pku.cn) database was employed (Tang et al., 2019). Supplementary Figure S1 illustrates the technical framework of this study.

### Identification of differentially expressed genes

We initially sourced the GSE31210_RAW and GSE31210_series_matrix.txt.gz files from the Gene Expression Omnibus (GEO) database (http://www.ncbi.nlm.nih.gov/geo). Utilizing the R package oligo, we meticulously processed the raw microarray data, executing a series of corrections that encompassed background subtraction, normalization, imputation of missing data points, and log2 transformation, thereby yielding a pristine gene expression matrix. Subsequently, employing the limma package, we conducted differential expression analysis, with a stringent criterion of |log2FC| > 1 and P value <0.05 to identify significantly differentially expressed genes.

### Identification of key gene modules

Gene expression profiling data from the GSE31210 cohort (n = 246 LUAD samples) were preprocessed to obtain a normalized expression matrix, with batch effects removed using Combat algorithm. We employed the WGCNA package (v1.72) in R to construct scale-free co-expression networks, with soft-thresholding power (β = 12) determined by scale-free topology criterion (*R* ^2^ > 0.85). Module detection was performed using dynamic tree cutting (minimum module size = 30 genes, merge CutHeight = 0.25), followed by Pearson correlation analysis between module eigengenes and tumor phenotype (|r|>0.5, FDR-adjusted p < 0.01). The turquoise module demonstrated the strongest association with malignant progression (r = 0.63, p = 3.2 × 10^−7^), and its constituent genes were subjected to downstream functional annotation. Detailed WGCNA parameters are documented in Supplementary Table S3, with methodological references to Langfelder & Horvath (Langfelder and Horvath, 2008).

### Functional and pathway enrichment analysis

To investigate the biological processes linked to these key genes, we performed Gene Set Enrichment Analysis (GSEA) using the “GSEA” R package, revealing mechanisms associated with the identified DEGs. Additionally, we conducted Gene Ontology (GO), Kyoto Encyclopedia of Genes and Genomes (KEGG) pathway analysis, and Disease Ontology (DO) enrichment analyses using “clusterProfiler”. Furthermore, we employed Single Sample Gene Set Enrichment Analysis (ssGSEA) using the GSVA package to assess the enrichment of Hallmark gene sets from MSigDB in each sample. Enriched functions and pathways were visually represented with “ggplot2”, considering an adjusted P-value <0.05 as significant.

### Protein-protein interaction network and hub gene identification

Using the Search Tool for the Retrieval of Interacting Genes (STRING; https://string-db.org), which aggregates experimental and computationally predicted interactions from 12,535 organisms encompassing 59.3 million proteins and over 20 billion interactions, we systematically constructed a protein-protein interaction (PPI) network for target genes. All interactions were filtered with a medium-confidence threshold (score ≥0.400) to ensure biological relevance.

The resulting network was imported into Cytoscape software (v3.8.2) for visualization and topological analysis. Functional modules were subsequently extracted using the Molecular Complex Detection (MCODE) plugin (degree cutoff = 2, node score cutoff = 0.2, K-core = 2, max depth = 100) to identify densely interconnected hub genes with potential biological significance.

### Machine learning analysis

Hub genes from the PPI network were further screened as LUAD diagnostic biomarkers using three machine learning algorithms: LASSO (glmnet, family = “binomial”, alpha = 1 with 10-fold cross-validation to select the optimal λ), Random Forest (randomForest with ntree = [500/1000] and variable importance evaluation), and SVM-RFE (e1071 + caret using repeated cross-validation) (Feng et al., 2023; Kumar et al., 2017; Savargiv et al., 2021). Genes consistently selected by all three methods (LASSO&RF&SVM-RFE) were defined as consensus diagnostic hub genes. Model-specific importance/ranking metrics (LASSO coefficients, RF importance, and SVM-RFE ranks) are provided in Supplementary Table S2. Diagnostic performance of each candidate gene was assessed by ROC analysis using pROC; the AUC and its 95% confidence interval (DeLong) were calculated, and AUC >0.7 was considered indicative of good diagnostic value.

### Survival analysis

Survival implications were interrogated using PrognoScan (http://www.prognoscan.org) and GEPIA2 (http://gepia2.cancer-pku.cn) (Tang et al., 2019). PrognoScan mines public cancer microarray repositories to relate gene-expression levels to prognosis (Mizuno et al., 2009); GEPIA2 standardizes TCGA and GTEx data to generate OS curves via its built-in Survival module. Hazard ratios with 95% confidence intervals and log-rank P values were extracted directly from each platform. Specifically, Overall survival analyses were conducted using Cox proportional hazards models. Patients were stratified into high and low EMCN expression groups using the median expression as the cutoff. Hazard ratios were reported as HR_ {high vs. low}, where HR < 1 indicates a protective association for the high-expression group.

### Immune infiltration analysis

Immune infiltration was quantified with TIMER2.0 (http://timer.cistrome.org), which deconvolves RNA-seq data while correcting for tissue-specific expression to yield accurate abundance estimates of six major immune subsets (B cells, CD8^+^ and CD4^+^ T cells, neutrophils, macrophages and dendritic cells). EMCN expression versus immune-cell levels in LUAD was computed using the built-in “Gene” module; Spearman ρ and associated P values were downloaded directly (Li et al., 2020).

### Immunohistochemistry

Tumor tissues were fixed in 4% paraformaldehyde (PFA) for 24 h, embedded in paraffin, and sectioned into 4 μm thick slices. These sections were dewaxed, quenched for endogenous peroxidase activity, and blocked with normal goat serum. After 1 h of blocking, the sections were incubated with an anti-EMCN antibody (1:1000 dilution, PA5-21395, Thermo Fisher Scientific) according to the manufacturer’s instructions, followed by incubation with a secondary antibody. Fresh DAB solution was applied to each section. Finally, the slides were counterstained with hematoxylin and coverslipped. Images were captured using a Pannoramic scanner (3DHistech, Hungary). All the human tissue samples we used have been reviewed by the Biomedical Research Ethics Committee of the Second Affiliated Hospital of Nanchang University (Approval No.: Medical Research Ethics Review [2024] No. (82)).

The Human Protein Atlas (HPA, https://www.proteinatlas.org) database was also used to immunohistochemical (IHC) analysis (Karlsson et al., 2021). This analysis served to validate the protein expression levels of EMCN, which we specifically selected from both normal and LUAD tissues.

### Western blot and quantitative real-time PCR

Equal amounts of boiled lysates were resolved on 10% SDS-PAGE (PG112, Epizyme), transferred to methanol-activated PVDF membranes, blocked with 5% non-fat milk (1 h, RT) and probed with primary antibodies (4 °C, overnight). HRP-conjugated secondary antibodies (1 h, RT) were detected by enhanced chemiluminescence (SB-WB004, Sharebio); band intensities were quantified with ImageJ and normalized to β-actin.

Total RNA was isolated with TRIzol (15,596,026, Thermo Fisher) and an RNA extraction kit (AG21017, Accurate Biology), reverse-transcribed with kit RK20429 (ABclonal), and amplified on a 7900HT Fast Real-Time PCR System using SYBR Green (AG11739, Accurate Biology). Relative EMCN mRNA levels were calculated by the 2^−ΔΔCT^ method with 18S rRNA as endogenous control.

## Results

### Key genes screening and diagnostic model construction

**FIGURE 1:**
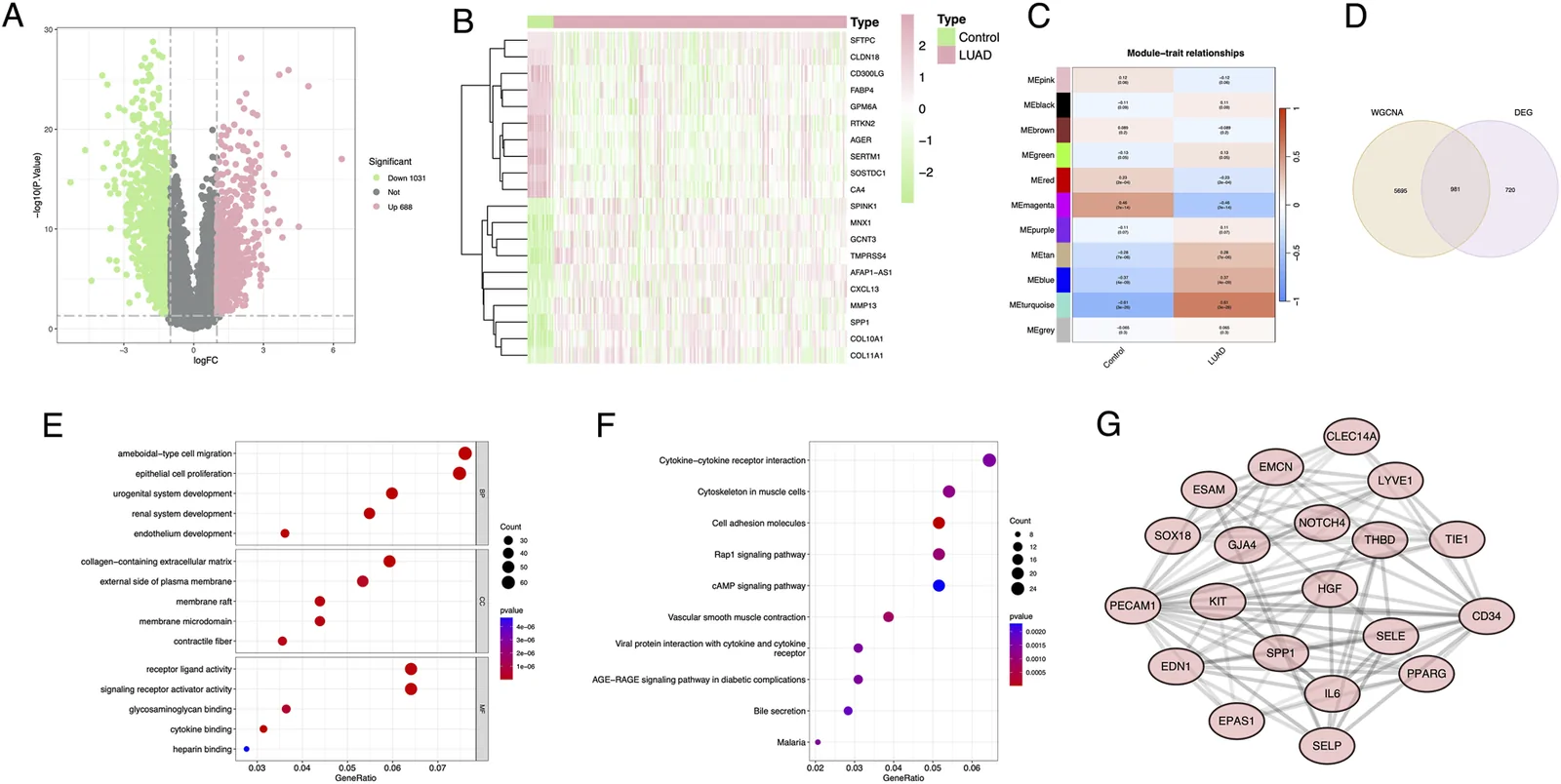
Transcriptome difference analysis and WGCNA analysis. **(A)** The volcano of different expression genes; **(B)** The heatmap of top 20 significant up and downregulate genes; **(C)** The relationship of each gene modules and group; **(D)** Veen diagram of significant differential expression and MEturquoise module; **(E)** and **(F)** shows the GO and KEGG enrichment results; **(G)** The gene network of hub genes in protein-protein interaction analysis of overlap genes.

Differential expression analysis of the GSE31210 dataset using DESeq2 (threshold: |log_2_FoldChange| > 1, adjusted p-value <0.05) identified 1,701 significantly dysregulated genes, including 1,013 downregulated and 688 upregulated (Figure 1A; Supplementary Table S1). Top 20 differentially expressed genes (DEGs) are visualized in Figure 1B. Weighted gene co-expression network analysis (WGCNA) revealed the turquoise module as the most strongly correlated with tumor phenotype (r = 0.61, p = 3 × 10^−26^; Figure 1C), containing 981 overlapping DEGs (Figure 1D). Functional enrichment analysis demonstrated significant associations with epithelial cell proliferation and activation of cytokine-cytokine receptor interaction and Rap1/cAMP signaling pathways (Figures 1E,F). Protein-protein interaction network analysis via STRING database with MCODE clustering (score cutoff ≥4) identified EMCN among core hub genes (Figure 1G).

**FIGURE 2:**
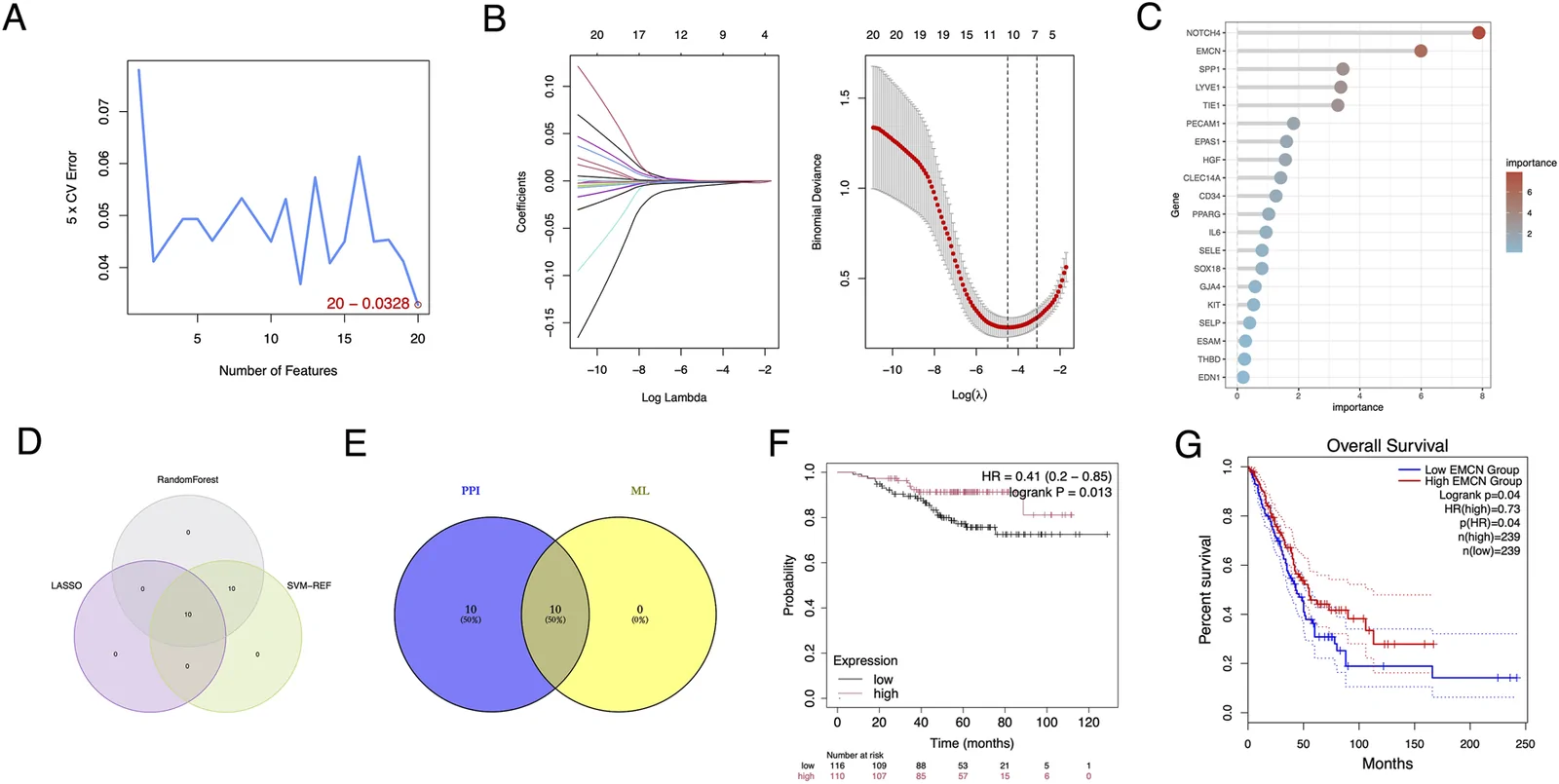
**(A)** Identification of signature genes from hub genes using the SVM-RFE algorithm; **(B)** Identification of signature genes using the LASSO logistic regression algorithm; **(C)** Identification of signature genes using the Random Forest (RF) algorithm; **(D)** Veen diagram of key genes screened by three machine learning algorithms; **(E)** Veen diagram of intersection between PPI hub genes and 10 selected genes in Figure 2D; **(F)** and **(G)** Survival curves of EMCN expression and patient survival based on KMplot and GEPIA2 database.

To systematically validate these candidates, we employed three machine learning algorithms: Support vector machine-recursive feature elimination (SVM-RFE) selected 20 signature genes (Figure 2A; Supplementary Table S2). LASSO regression (λ = 0.1, 10-fold cross-validation) identified 10 prognostic markers (Figure 2B); Random Forest analysis ranked top 20 features by Gini index (Figure 2C). Intersection analysis confirmed 10 consensus genes shared across all methods (Figure 2D), which were further cross-validated with WGCNA-derived hub genes to establish the final candidate list (Figure 2E). Machine-learning screening with three algorithms yielded candidate genes listed in Supplementary Table S2, and their expression between LUAD and normal tissues were further characterized (Supplementary Figure S2). Using the median EMCN expression as the cutoff, Kaplan–Meier and Cox proportional hazards analyses indicated that patients with high EMCN expression had better overall survival than those with low expression (HR_{high vs. low} = 0.73, p = 0.04; Figures 2F,G). Thus, low EMCN expression is associated with poorer prognosis in LUAD, consistent with the adverse prognosis associated with reduced CD34, THBD and NOTCH4 expression (Supplementary Figure S3A). Notably, elevated EDN1 and GJA4 expression also correlated with worse clinical outcomes (adjusted p < 0.05; Supplementary Figure S3B).

### Molecular characterization of EMCN

**FIGURE 3:**
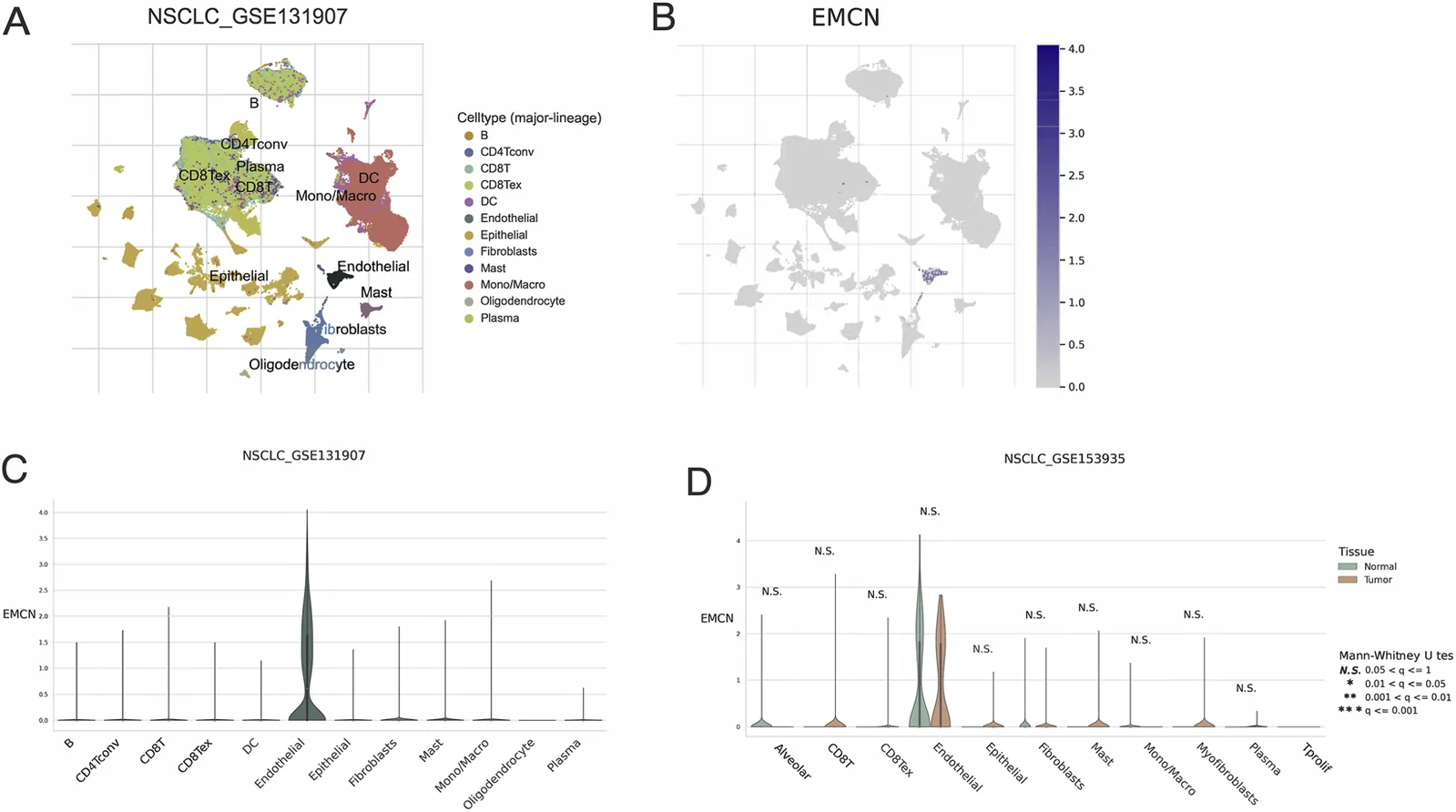
The single cell RNAseq of NSCLC. **(A)** The UMAP of cell type annotation; **(B)** Feature plot of EMCN expression; **(C)** The violin plot of EMCN expression in each cell types; **(D)** The violin plot of EMCN expression in Endothelial between LUAD group and control group.

To delineate the expression pattern of EMCN within tumor microenvironments, we analyzed NSCLC single-cell RNA-seq data from TISCH2 (http://tisch.comp-genomics.org/home/). EMCN exhibited predominant expression in endothelial cells, with negligible expression detected in epithelial malignant cells or other non-endothelial compartments (Figures 3A–C). This endothelial-enriched pattern is consistent with the known biology of Endomucin and supports interpreting EMCN as a vascular-associated marker in LUAD rather than a tumor epithelial marker. Although comparative analysis revealed marginally higher EMCN levels in normal versus tumor-associated endothelia, this difference lacked statistical significance (p > 0.05, two-tailed t-test, Figure 3D).

**FIGURE 4:**
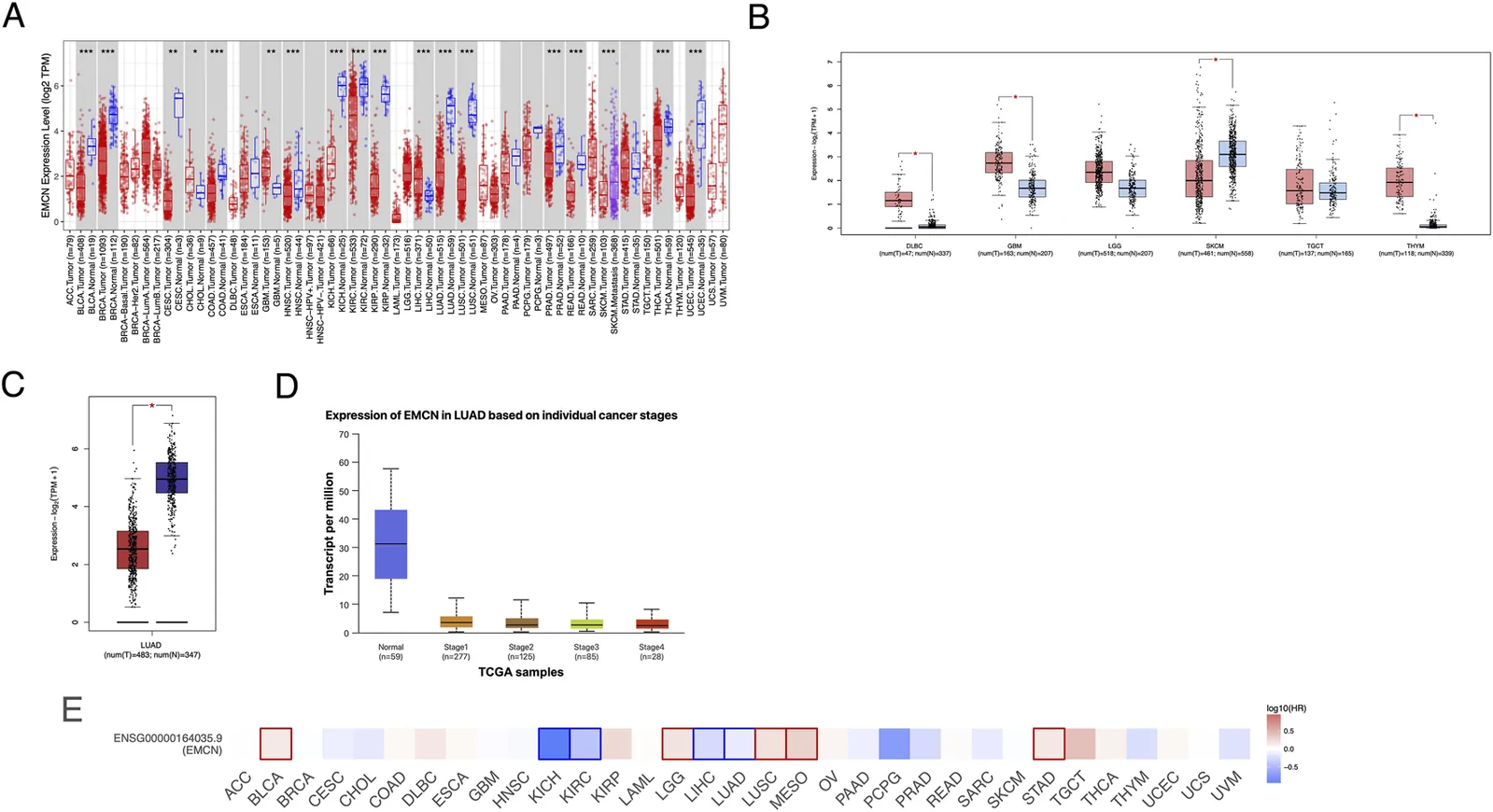
The expression profile of EMCN. **(A)** The expression of EMCN in pan-cancer across the TCGA database analyzed by TIMER2; **(B)** Differential expression of EMCN in DLBC, GBM, LGG, SKCM, TGCT and THYM.; **(C)** Verification of EMCN expression using GEPIA2; **(D)** Exploration of EMCN expression differences in each stage of LUAD using GEPIA2; **(E)** OS analysis of EMCN across TCGA cancers. The figure was also constructed using GEPIA2, with the red line representing high expression of EMCN and the blue line representing low expression of EMCN. *p < 0.05, **p < 0.01 and ***p < 0.001. Note: ACC, adrenocortical carcinoma; BLCA, bladder urothelial carcinoma; BRCA, breast invasive carcinoma; BRCA-Basal, breast invasive carcinoma-basal; BRCA-Luminal, breast invasive carcinoma-luminal; BRCA-Her2, breast invasive carcinoma-her2; CESC, cervical and endocervical cancer; CHOL, cholangiocarcinoma; COAD, colon adenocarcinoma; DLBC, diffuse large B-cell lymphoma; ESCA, esophageal carcinoma; GBM, glioblastoma multiforme; HNSC, head and neck cancer; HNSC-HPV+, head and neck cancer-HPV positive; HNSC-HPV−, head and neck cancer-HPV negative; KICH, kidney chromophobe; KIRC, kidney renal clear cell carcinoma; KIRP, kidney renal papillary cell carcinoma; LGG, lower grade glioma; LIHC, liver hepatocellular carcinoma; LUAD, lung adenocarcinoma; LUSC, lung squamous cell carcinoma; MESO, mesothelioma; OV, ovarian serous cystadenocarcinoma; PAAD, pancreatic adenocarcinoma; PCPG, pheochromocytoma and paraganglioma; PRAD, prostate adenocarcinoma; READ, rectum adenocarcinoma; SARC, sarcoma; SKCM, skin cutaneous melanoma; SKCM-Primary, skin cutaneous melanoma-primary; SKCM-Metastasis, skin cutaneous melanoma-metastasis; STAD, stomach adenocarcinoma; TGCT, testicular germ cell tumors; THCA, thyroid carcinoma; THYM, thymoma; UCEC, uterine corpus endometrial carcinoma; UCS, uterine carcinosarcoma; UVM, uveal melanoma.

Pan-cancer analysis via TIMER2.0 demonstrated differential EMCN expression across 18 malignancies (FDR <0.05, Figure 4A), showing upregulated patterns in BLCA and BRCA among 15 cancer types, contrasted by downregulation in GBM and LIHC. Integration of GTEx v8 and TCGA v32 datasets confirmed elevated EMCN expression in DLBC and THYM (Figure 4B). TCGA analysis corroborated significant EMCN suppression in LUAD tumors versus adjacent normal tissue (p < 0.05; Figure 4C), consistent with GSE31210 findings. Stage-stratified analysis revealed persistently reduced EMCN across all tumor stages compared to normal (p < 0.001, Figure 4D), though inter-stage variations were nonsignificant (Supplementary Figure S4).

Prognostic assessment using GEPIA2 uncovered cancer-type-specific survival associations: EMCN expression negatively correlated with OS in KICH (HR = 1.67, p = 0.03) and KIRC (HR = 1.45, p = 0.04), yet conferred protective effects in BLCA (HR = 0.62, p = 0.008) and LGG (HR = 0.59, p = 0.003) (Figure 4E).

### Vascular-immune regulatory mechanisms

**FIGURE 5:**
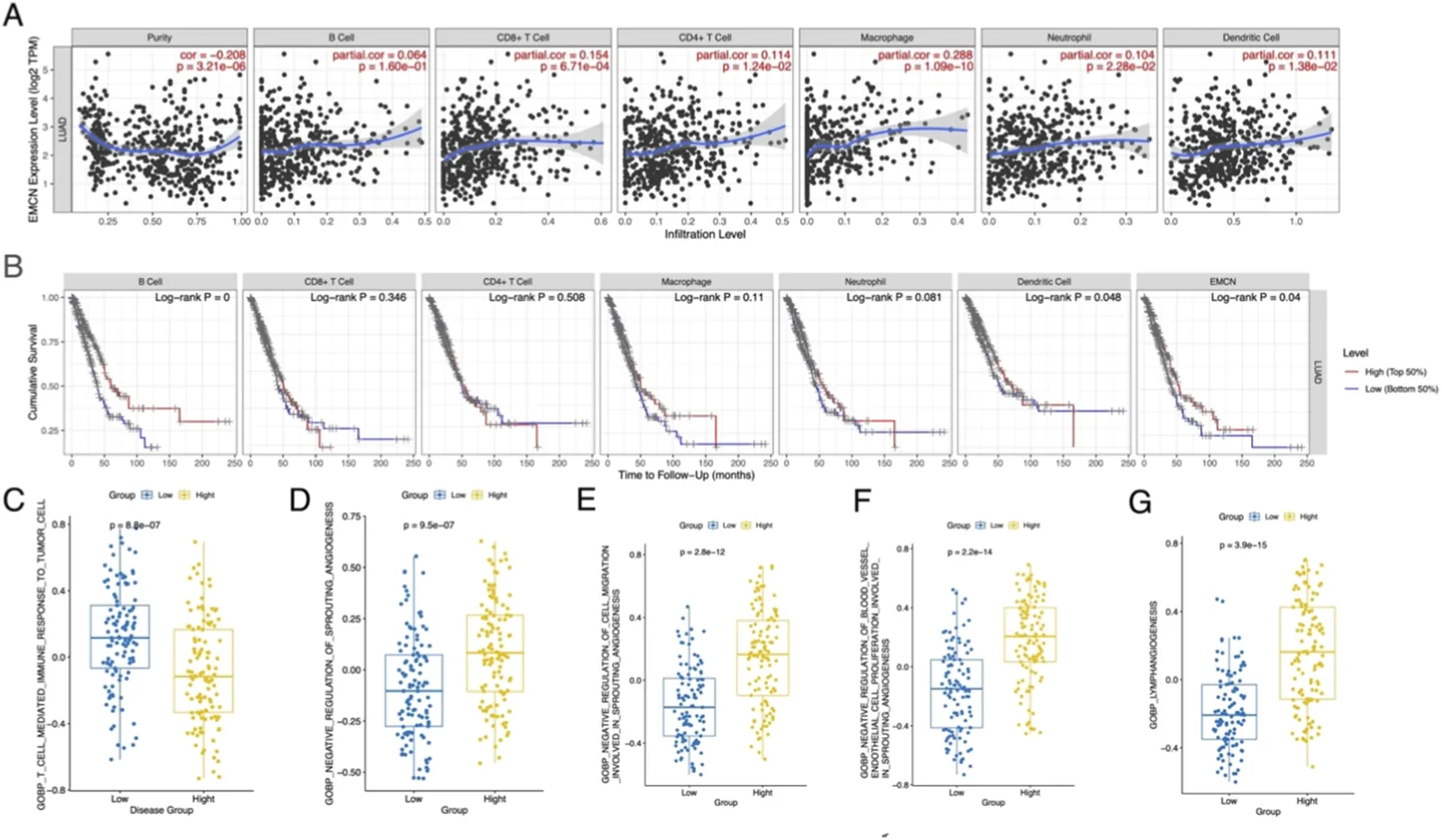
Analysis of immune infiltration and functional enrichment using ssGSEA. **(A)** The correlation analysis of EMCN expression and immune cell-types using TIMER2 database; **(B)** The survival analysis of EMCN and immune cell-types in LUAD; **(C–G)** The boxplot of ssGSEA enrichment in GOBP_T_CELL_MEDIATED_IMMUNE_RESPONSE_TO_TUMOR_CELL, GOBP_NEGATIVE_REGULATION_OF_SPROUTING_ANGIOGENESIS, TIVE_REGULATION_OF_CELL_MIGRATION_INVOLVED_IN_SPROUTING_ANGIOGENESIS, TION_OF_BLOOD_VESSEL_ENDOTHELIAL_CELL_PROLIFERATION_INVOLVED_IN_SPR, GOBP_LYMPHANGIOGENESIS.

**FIGURE 6:**
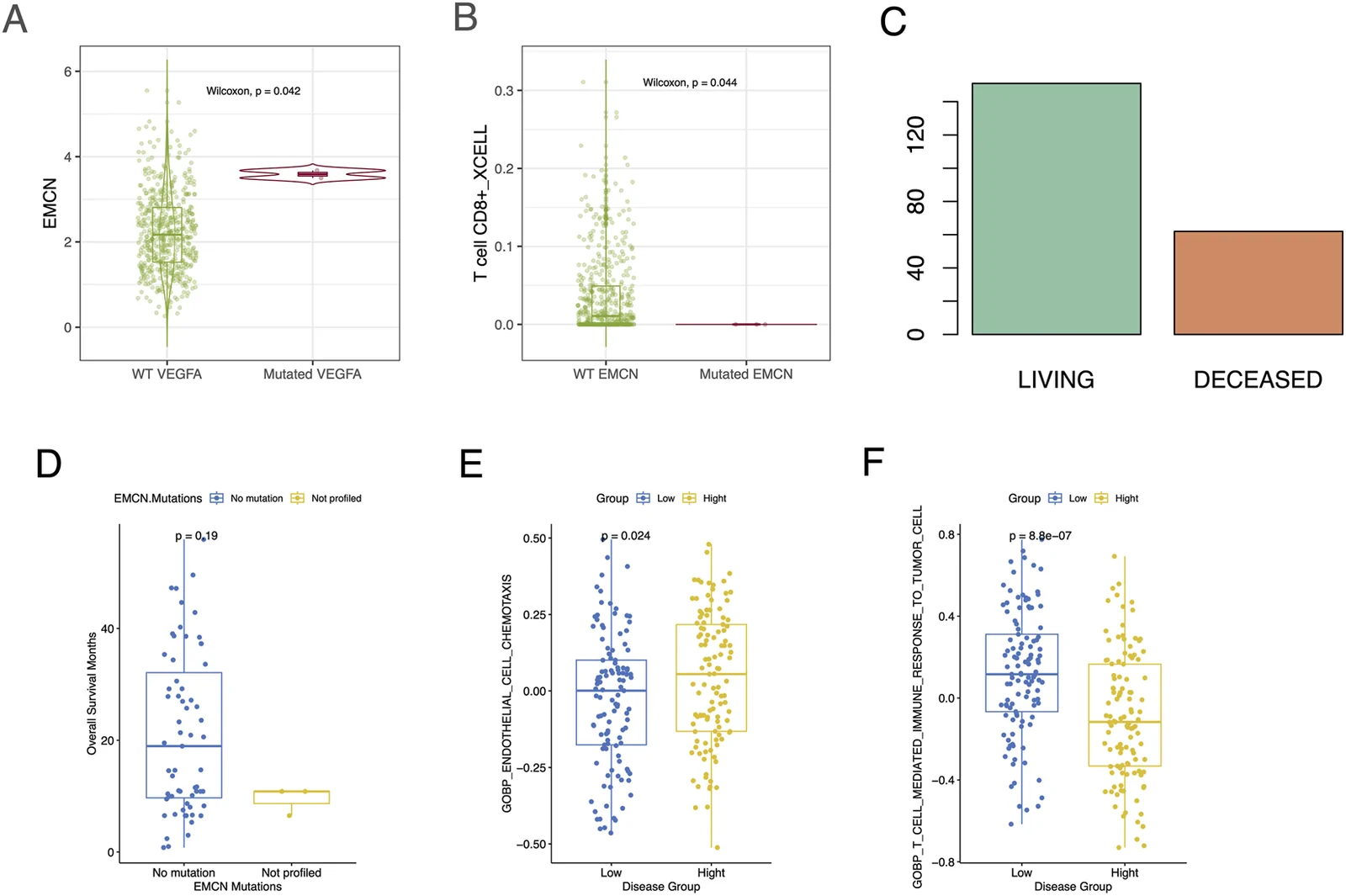
The function of EMCN in angiogenesis. **(A)** Violin plot of EMCN expression between WEGFA mutation group and wild type group in LUAD; **(B)** Violin plot of CD8^+^ T cells abundance between EMCN mutation group and wild type group in LUAD; **(C)** The number of living and death patient in EMCN wild type group. **(D)** The overall survival month of EMCN wild type patient; **(E,F)** The boxplot of ssGSEA enrichment of GOBP_ENDOTHELIAL_CELL_CHEMOTAXIS and GOBP_T_CELL_MEDIATED_IMMUNE_RESPONSE_TO_TUMOR_CELL signaling pathway between EMCN high expression group and low expression group.

Comprehensive analysis of TCGA-LUAD data via TIMER2.0 revealed significant positive correlations between EMCN expression and infiltration levels of CD8^+^ T cells (r = 0.154, p = 6.71 × 10^−4^), macrophages (r = 0.288, p = 1.09 × 10^−10^), and dendritic cells (r = 0.111, p = 1.38 × 10^−2^), whereas no association was observed with B cells (p = 0.16) (Figure 5A). Survival analysis demonstrated improved prognosis in patients with high B-cell infiltration but reduced survival with elevated dendritic cell presence (p = 0.048) (Figure 5B). Intriguingly, EMCN low tumors exhibited enhanced activity of GOBP_T_CELL_MEDIATED_IMMUNE_RESPONSE_TO_TUMOR_CELL (NES = 1.9, FDR = 0.03), suggesting compensatory immune activation in EMCN-deficient microenvironments (Figure 5C). Furthermore, ssGSEA analysis revealed differential enrichment of the GOBP_T_CELL_MEDIATED_IMMUNE_RESPONSE_TO_TUMOR_CELL signaling pathway between EMCN high and low expression groups, with distinct immune response patterns observed between the two groups (Figure 6F). In addition, we found that high expression of EMCN significantly enriched the GOBP_LYMPHANGIOGENESIS signaling pathway (Figure 5G), which may indicate the involvement of EMCN in immune bidirectional regulation. Functional enrichment analysis using ssGSEA identified significant suppression of angiogenic pathways in EMCN low tumor group, including: GOBP_NEGATIVE_REGULATION_OF_SPROUTING_ANGIOGENESIS, GOBP_NEGATIVE_REGULATION_OF_CELL_MIGRATION_INVOLVED_IN_SPROUTING_ANGIOGENESIS, GOBP_NEGATIVE_REGULATION_OF_BLOOD_VESSEL_ENDOTHELIAL_CELL_PROLIFERATION_INVOLVED_IN_SPROUTING_ANGIOGENESIS (Figures 5D–F).

Conversely, EMCN high group demonstrated enrichment of GOBP_ENDOTHELIAL_CELL_CHEMOTAXIS, indicating its role in maintaining vascular homeostasis (Figure 6E). Mutation analysis revealed elevated EMCN expression in VEGFA-mutant tumors (p = 0.042) (Figure 6A). Patients with wild-type EMCN exhibited higher CD8^+^ T-cell infiltration (p = 0.044, Figure 6B) and numerically longer median OS in the Lung Adenocarcinoma (CPTAC GDC, 2025) cohort (Figures 6C,D).

### Clinical translational validation

**FIGURE 7:**
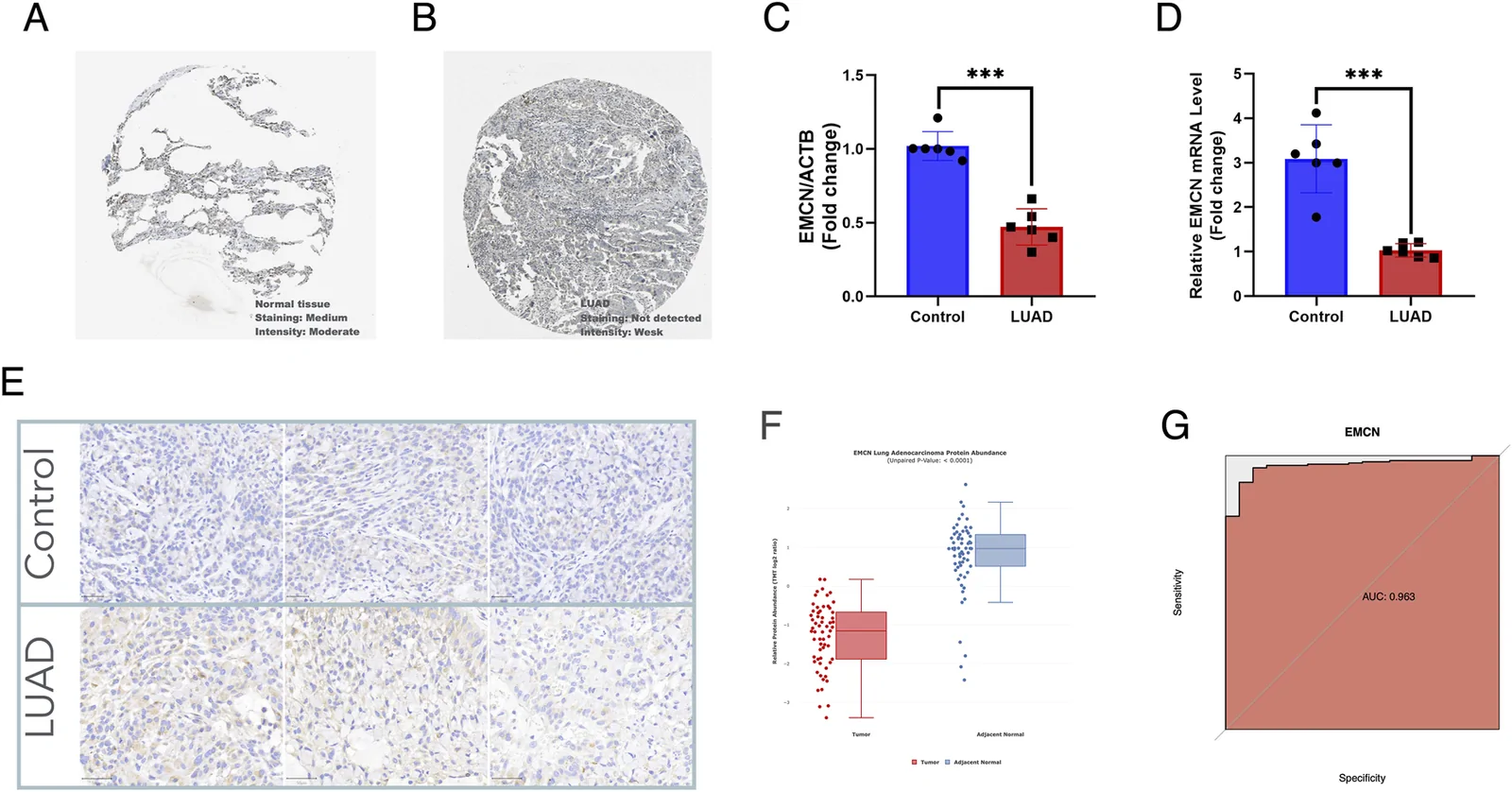
Validation and diagnostic value of EMCN. **(A,B)** Comparison of immunohistochemistry images showing EMCN protein expression between normal (left) and tumor tissues in the Human Protein Atlas (HPA) dataset. **(C)** Validation of EMCN protein expression by Western blotting and corresponding statistical analysis. **(D)** Detection of relative EMCN mRNA expression levels in the LUAD and control groups using qRT-PCR. **(E)** Representative immunohistochemistry images and quantitative analysis of EMCN staining in the institutional LUAD cohort and adjacent normal tissues. Scale bar: 50 μμm. **(F)** Proteomic analysis of EMCN protein expression in LUAD specimens from the CPTAC dataset. **(G)** Receiver operating characteristic (ROC) curve analysis of EMCN diagnostic performance in the GSE31210 dataset. *p < 0.05, **p < 0.01, ***p < 0.001.

Proteomic analysis of CPTAC v3.2 demonstrated significant downregulation of EMCN protein in LUAD specimens (Figure 7F), a finding corroborated by immunohistochemistry (IHC) data from the Human Protein Atlas (Figures 7A,B). Independent validation using our institutional LUAD cohort (n = 98) confirmed consistent EMCN suppression in tumor tissues (IHC intensity score 1.7 ± 0.3 vs. adjacent normal 3.2 ± 0.4, p = 0.002; Figure 7E).

Western blot and qRT-PCR analyses revealed concordant reduction at both protein (p < 0.05) and mRNA levels (p < 0.05) in LUAD compared to controls (Figures 7C,D). Receiver operating characteristic (ROC) curve analysis in GSE31210 cohort established EMCN’s exceptional diagnostic performance (AUC = 0.963, 95% CI 0.942–0.984), with sensitivity of 92.1% and specificity of 89.7% at optimal cutoff (Figure 7G).

## Discussion

Through integrative analysis of large-scale LUAD cohort data employing co-expression networks and machine learning algorithms, EMCN was identified as a vascular-associated gene exhibiting paradoxical survival benefits in low-expression subgroups. Single-cell RNA sequencing revealed endothelial-specific EMCN expression, with a non-significant trend toward higher levels in control versus tumor tissues - a pattern concordant with bulk RNA-seq findings. Notably, EMCN expression demonstrated significant positive correlations with tumor-infiltrating immune cells (CD8^+^ T cells: r = 0.38, p = 0.008; CD4^+^ T cells: r = 0.29, p = 0.02; macrophages: r = 0.31, p = 0.01; Figure 5A).

Consistent with these observations, immune-related pathways were enriched in EMCN low group (FDR<0.05, Figure 5C), suggesting dual functionality in both angiogenesis and immunomodulation. Given the endothelial-restricted expression of EMCN observed in scRNA-seq, the reduced EMCN expression in bulk LUAD tumors may reflect changes in the vascular compartment, including altered endothelial abundance and/or endothelial transcriptional reprogramming within the tumor microenvironment. Therefore, EMCN should be interpreted primarily as a vascular-associated feature linked to tumor–stroma–immune interactions rather than a direct epithelial tumor-cell marker. Diagnostic validation through immunohistochemistry and ROC analysis confirmed EMCN’s clinical utility (AUC = 0.963), indicating that EMCN can serve as a promising prognostic biomarker for LUAD.

The specific expression pattern of EMCN in endothelial cells, coupled with its pan-cancer dysregulation, suggests a tissue-specific yet evolutionarily conserved mechanism governing tumor vascularization. The EMCN gene encodes a transmembrane O-sialylated protein predominantly expressed on endothelial cell surfaces, where it participates in angiogenesis. *In vivo* knockdown studies demonstrate that EMCN deficiency impairs vascular development in the retina and significantly attenuates VEGF-induced endothelial, tube formation, and proliferation *in vitro* (Park-Windhol et al., 2017; Hu et al., 2025). Intriguingly, our study reveals EMCN accompanied by enrichment of angiogenesis-suppressive pathways GOBP_NEGATIVE_REGULATION_OF_SPROUTING_ANGIOGENESIS, etc., Figures 5D–F) in EMCN-high group, suggesting the negative regulatory mechanism of EMCN. However, EMCN appears to facilitate vascular normalization, as evidenced by significant enrichment of the GOBP_ENDOTHELIAL_CELL_CHEMOTAXIS pathway (Figure 6E) - a critical mediator of neovascularization. This dual functionality is further corroborated by reduced EMCN expression in VEGFA-mutant tumors (Figure 6A), aligning with its putative angiogenesis-modulatory role. Immunologically, EMCN expression positively correlates with infiltration of CD8^+^ T cells (r = 0.154, p < 0.01), CD4^+^ T cells, and macrophages in LUAD specimens. This immunomodulatory capacity, coupled with its association with favorable patient outcomes (HR = 0.73, p = 0.04), suggests EMCN may enhance antitumor immunity through vascular-mediated immune cell trafficking.

Beyond mechanistic insights, our clinical validation cohort reveals EMCN’s translational potential as evidenced by its exceptional diagnostic accuracy (AUC >0.96) and independent prognostic value (HR = 1.89). Notably, survival analyses consistently suggested that higher EMCN expression is protective, as reflected by HR_{high vs. low} = 0.73 (p = 0.04), implying that EMCN downregulation is associated with worse overall survival. These findings align with the work by Dai et al. demonstrating EMCN’s prognostic value in gastrointestinal cancers, where an EMCN/MUC15 signature emerged as a promising biomarker (Dai et al., 2020). The conserved tumor-suppressive role of EMCN across malignancies strengthens its candidacy as a pan-cancer biomarker worthy of further clinical exploration.

While this study provides comprehensive evidence for the multifaceted functions of Endomucin (EMCN), several limitations should be acknowledged regarding spatial resolution and causal validation. First, our study did not incorporate spatial transcriptomic data, leaving the expression patterns of EMCN in different regions of lung adenocarcinoma (LUAD) patients unknown. Second, due to funding constraints and time limitation, although our analyses support an association between EMCN and the vascular-immune contexture of LUAD, we did not perform endothelial cell-based experimental validation (e.g., in HPMEC or HUVEC) to directly test mechanistic hypotheses. We did not conduct gene knockout studies to construct animal models, and thus lack *in vivo* functional validation of EMCN. However, the primary objective of this study was to explore the diagnostic value of EMCN for LUAD, and we consider these limitations forgivable in the context of our research goals. If this article is accepted for publication by our peers, we will continue to delve into the study of EMCN, future work will incorporate endothelial models and functional perturbation experiments to clarify how EMCN-related endothelial programs may influence immune infiltration and clinical outcomes. First, we will supplement our research with a conditional knockout mouse model of EMCN and investigate its functions and phenotypic analysis *in vivo*. Additionally, we will explore the expression patterns of EMCN in body fluids such as plasma or bronchoalveolar lavage fluid from LUAD patients to prospectively validate the value of liquid biopsy. Moreover, we will thoroughly investigate the methylation regulation of EMCN and explore whether the methylation of EMCN, in combination with other clinical indicators, can be used for the early screening of LUAD.

In conclusion, we propose the EMCN signature as a prognostic indicator driven by coordinated angiogenic and immunomodulatory mechanisms. It predicts LUAD prognosis and supports future evaluation of EMCN as a biomarker for patient stratification and liquid biopsy.

## Data Availability

Publicly available datasets were analyzed in this study. This data can be found here: TCGA-LUAD, GSE31210 and Lung Adenocarcinoma (CPTAC GDC, 2025).
